# Like parent, like child: a cross-sectional study of intra-household consumption patterns of non-alcoholic beverages among British households with children

**DOI:** 10.1017/S1368980021005061

**Published:** 2022-07

**Authors:** Charlotte O’Leary, Steven Cummins, Richard D Smith, Laura Cornelsen

**Affiliations:** 1 Faculty of Public Health and Policy, London School of Hygiene & Tropical Medicine, Keppel Street, London WC1E 7HT, UK; 2 Royal Melbourne Hospital, Melbourne, Australia; 3 College of Medicine and Health, University of Exeter, Exeter, UK

**Keywords:** Intra-household consumption, Great Britain, Sugar-sweetened beverages, Non-alcoholic beverages, Children

## Abstract

**Objective:**

Most research investigating sugar-sweetened beverages (SSB) and health, conducted at the individual or household level, ignores potentially important intra-household dynamics. We analysed self-reported consumption relationships between children and adults, and between children of different ages, as well as the associations between intra-household consumption, BMI and sociodemographic characteristics.

**Design:**

A cross-sectional analysis of survey data from Kantar Fast Moving Consumer Goods panellists in September 2017.

**Setting:**

Great Britain.

**Participants:**

Random sample of 603 households with children under 18 years who regularly purchase non-alcoholic beverages.

**Results:**

Low- or no-sugar/diet beverages dominate consumption across all age categories, particularly children under 12 years. SSB consumption increased as children became older. Children’s reported consumption of SSB and low- or no-sugar/diet beverages was positively associated with consumption by adults; a child in adolescence had over nine times the odds of consuming SSB (adjusted OR 9·55, (95 % CI 5·38, 17·00), *P* < 0·001), and eight times the odds of consuming low- or no-sugar/diet drinks (adjusted OR 8·12, (95 % CI 4·71, 13·97), *P* < 0·001), if adults did so. In households with multiple children, consumption patterns of older siblings were associated with those of the younger; notably a perfect correlation between children aged 0 and 6 years consuming SSB if siblings 13–18 years did so, and children aged 7–12 years had 22 times the odds of consuming SSB if siblings aged 13–18 years did so (OR 22·33, (95 % CI 8·60, 58·01), *P* < 0·001).

**Conclusions:**

Multiple policies, targeting children as well as adults, such as fiscal levers and advertisement restrictions, are needed to reduce and prevent the consumption of SSB.

Non-communicable diseases are a growing global health threat^(1)^. The excess consumption of dietary sugar, and the contribution of sugar-sweetened beverages (SSB) to this excess, have received much international attention in the search for modifiable risk factors for non-communicable disease prevention. In the UK, mean intake of sugar exceeds the maximum recommended^(2)^ in all age groups^(3)^, and non-alcoholic beverages are the second highest contributor to sugar intake in both adults and children^(3)^. Similar patterns are seen across a number of countries, with the affordability and accessibility of SSB increasing globally over the last few decades^(4)^.

Globally, increasing attention is being placed on fiscal policies to reduce the consumption of unhealthy foods and beverages, particularly SSB^(5)^. Price policies have the potential to generate revenue, reduce consumption and correct negative externalities of unhealthy consumption^(6,7)^. Taxes and levies on SSB have been implemented in over forty countries^(8)^. In April 2018, the UK government implemented the Soft Drinks Industry Levy^(9)^, a three-tiered levy which has encouraged significant product reformulation^(10)^ and generated revenue that is hypothecated for health spending^(11)^.

Fiscal policies are an attractive option for reducing consumption; however, factors that influence purchase and consumption are complex and can vary across the life course and by sociodemographic background^(12)^. Beverages can be consumed in a variety of settings, including in the home, at school/work, at social occasions and when eating out. Consistent with the social-ecological model of health promotion^(13)^, understanding the social, cultural and physical factors that impact on the home food environment is important in addressing the challenges of obesity, especially for children^(14–20)^. Pre-adolescent children are highly susceptible to factors in the home environment, have little purchasing autonomy outside of the home and are rapidly developing food tastes and habits^(21)^.

An important area of study is the extent to which the food and beverage consumption patterns of adults influence those of children in their household. A number of studies have demonstrated a positive correlation between parental sugary drink intake and children’s sugary drink intake at varying ages, from as young as infancy in the USA, Australia, the Netherlands and Belgium^(18,22–27,28)^. While focusing on low-income groups is a common approach^(23,24,29)^, there is less published data that assess these household consumption patterns across a range of sociodemographic characteristics, and different types of non-alcoholic beverages. Most studies that describe beverage consumption within families assess one child with one parent^(22–25,27,28)^. There may be, however, important consumption patterns seen between children of different ages in the same household, and according to the overall size and composition of the household unit. Additionally, the impact of siblings on children’s consumption has not been well established in the literature. None of these associations or patterns, to our knowledge, have been explored thus far in Great Britain.

This study aimed to fill this gap and examine the intra-household consumption of non-alcoholic beverages amongst households in Great Britain, with a particular focus on SSB consumption and consumption relationships between different members of the household unit: between children and adults, and between children of different ages. The impact of sociodemographic variables on these consumption patterns was also investigated, as well as how different types of non-alcoholic beverages (including SSB, low- or no-sugar/diet drinks and pure fruit juices) may be consumed differently by members of the household unit.

## Methods

### Data source

Data were from a discrete choice experiment study^(30)^ conducted in September 2017 examining the effects of framing and signalling of sugary drink taxes on beverage choice. Available data included questions on self-reported consumption within the household, described below, and responses to these questions were utilised for the purposes of this study.

The survey was conducted online using a random sample of 603 households (encompassing 1344 adults and 1104 children) drawn from Kantar Fast Moving Consumer Goods (FMCG) panel of households in Great Britain. Sample size was based on the design of the choice experiment for detecting effect sizes for the coefficients of interest in the experimental study^(30)^. Annual panel size where the sample is drawn from is approximately 30 000 households, and the panel is nationally representative with respect to geographical region, age of the main shopper, household size and occupational socio-economic status^(30)^. Panellists are offered vouchers of small value for high street retailers and leisure activities as compensation for panel participation.

The study sample was restricted to households who had purchased at least 2 l of non-alcoholic beverages every month in the 6 months preceding the survey (based on home-scan purchases and determined by Kantar) and who had at least one child under the age of 18 years. Participants meeting these two criteria were then randomly selected for invitation into the study. Assuming a response rate of about 70 % the survey was made available for 780 households through a weblink. The single-use link to the survey was sent to the main shopper in the household as identified by Kantar and we assumed that it was the main shopper (respondent hereafter) who completed the survey including providing data on beverage consumption by their household members. The survey was conducted during a 10-d period in September 2017 by Kantar who collected the survey results and provided further sociodemographic data (household size, number of children, income bracket, region, highest qualification, tenure, occupational socio-economic status and self-reported height and weight of the main shopper). These sociodemographic data are collected annually^(30)^.

In the survey, respondents were asked to indicate the number of people in four age group categories (adult 18+ years, children aged 0–6, 7–12 and 13–18 years) in their household who consume beverages in four different categories: regular soft drink (fizzy, juice drink or squash); low-sugar soft drink (fizzy, juice drink or squash), 100 % fruit juice or smoothie; or water (still or sparkling)^1^. The question was worded as ‘Who in the household consumes the following drinks?’ with the respondent presented with the above-described four categories of beverages for each age group. A binary yes/no option was available for each response. Where households had more than one person in the respective age category, they were asked to specify how many of these individuals would consume these beverages. No data on quantities or regularity of this consumption were collected.

We focused analyses on three groups of beverages of public health interest (SSB, low or no-sugar/diet beverages, and pure fruit juice and smoothies), excluding bottled water. SSB and low or no-sugar/diet beverages include both fizzy (soda) drinks or juice drinks and concentrated drinks. SSB contain added sugar, low- or no-sugar/diet beverages are sweetened with non-caloric sweeteners, and pure fruit juice and smoothies contain naturally occurring sugar with no further sugar added.

The sociodemographic characteristics assessed in the study were household size, occupational SES in three categories (high, mid and low SES)^2^, household income^3^, region of residence^4^ and highest qualification^5^. BMI was calculated from self-reported height and weight^6^ and grouped into categories of healthy weight (BMI < 25), overweight (BMI 25–29·9) and obese (BMI 30 or above)^(31)^. Missing values were observed for income (*n* 86, 14·3 %), highest qualification (*n* 2, 0·33 %), height (*n* 45, 7·5 %) and weight (*n* 145, 24 %).

### Statistical analysis

We first conducted bivariate analyses, whereby self-reported consumption of each type of beverage by children or adults and overall was compared across the different sociodemographic strata. Chi-square tests for the difference in proportions were used to test the hypotheses that consumption patterns differed by sociodemographic strata.

To analyse adult–child consumption correlation, we calculated OR across the three drink categories and all child age groups. Stratified analyses were used to generate adjusted OR with each sociodemographic variable, then the test of homogeneity of OR was performed to determine the probability of interaction. Crude and fully adjusted OR of child consumption by adult consumption status were then estimated using logistic regression. Full adjustment was undertaken using variables with complete information for all respondents (*n* 603) and include SES, region, household size and age of the respondent. It is reasonable to assume that adult consumption primarily influences child consumption, particularly amongst younger children who have less autonomy in food and drink purchases, though it is possible that children may influence adult consumption, especially in older children^(32)^.

The association of having middle (aged 7–12 years) and older (aged 13–18 years) siblings who consumed beverages on young child (aged 0–6 years) beverage consumption and the association of having older siblings who consumed beverages on middle child beverage consumption were assessed through crude OR estimated with logistic regression. We did not estimate fully adjusted models for child–child consumption correlation because the sample size was not sufficient (deemed as greater than ten observations in each cell of the 2 × 2 table) to further investigate the sociodemographic characteristics using stratified analyses. All statistical analyses were conducted in Stata, version 16^(33)^.

## Results

The sample is described in Table 1. The majority of households (62·0 %) consisted of 4–5 people. A small proportion were single-parent households (7·0 %) and almost one in four households had more than two adults (23·8 %). A third of households had at least one child aged 0–6 years (32·3 %) and over half had at least one child aged 7–12 years (51·7 %), and one child aged 13–18 years (55·4 %). More than half of the households were in the middle SES group (51·7 %) and two-thirds earned more than £20 000 per year (68·8 %). Almost half of the sample respondents were aged 40–49 years (47·3 %). Of those who had provided their height and weight (75·5 % of the sample), 24·1 % were overweight and a further 19·4 % were obese. This is a lower prevalence of overweight and obesity than in the general UK population at the time of the survey – in 2016, 35 % of adults were overweight, and a further 29 % were obese^(34)^.

**Table 1:** Sociodemographic characteristics of the sample

Variable	Category	Number of households *n* 603	%
Household size	2–3 people	176	29·2
	4–5 people	374	62·0
	6+ people	53	8·8
Number of adults	1 adult in the family	42	7·0
	2 adults in the family	418	69·3
	3 adults in the family	112	18·6
	4 or 5 adults in the family	31	5·2
Presence of children	children aged 0–6 years	195	32·3
	children aged 7–12 years	312	51·7
	children aged 13–18 years	334	55·4
Socio-economic status of respondent*	High (AB)	102	16·9
	Middle (C1, C2)	348	57·7
	Low (DE)	153	25·4
Household income group (£)	0–19 999	114	18·9
	20 000–39 999	225	37·3
	40 000+	178	29·5
	Missing	86	14·3
Region	London/Anglia	136	22·6
	South/South West	85	14·1
	North/Yorkshire/Lancashire	177	29·4
	Midlands/Wales and West	159	26·4
	Scotland	46	7·6
Highest qualification of respondent	Degree or higher	156	25·9
	Higher education	109	18·1
	High school (A Level)	106	17·6
	High school (GCSE)	175	29·0
	Other/none	55	9·1
	Missing	2	0·33
Age of respondent	<40 years	163	27·0
	40–49 years	285	47·3
	50+ years	155	25·7
BMI of respondent	BMI < 25†	193	32·0
	Overweight 25–29·9	145	24·1
	Obese 30+	117	19·4
	Missing	148	24·5

* Based on employment category: AB = higher and intermediate managerial, administrative, professional occupations, C1 = supervisory, clerical and junior managerial, C2 = skilled manual occupations, D = semi-skilled and unskilled manual occupations, E = unemployed and lowest grade occupations.

† Six respondents (1·0 %) had a BMI of less than 18·5, which is considered underweight. For ease of analysis, we grouped these observations with those in the healthy range of BMI 18·5–25.

**Fig. 1:**
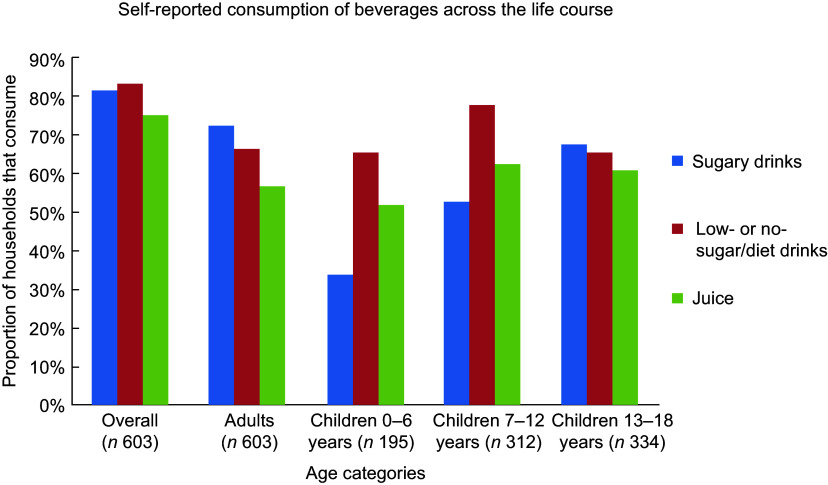
Self-reported consumption of beverages across the age groups. Presented as a proportion of households (with children in that age group, where appropriate) that consume each beverage

Figure 1 shows overall consumption by beverage type. Low- or no-sugar/diet drinks were consumed by the greatest proportion of households (83·6 %), and juice was consumed by the smallest proportion of households (74·8 %). Sugary drinks consumption increased markedly by age rising from 33·8 % of 0–6-year-olds to 67·7 % of 13–18-year-olds. By adulthood, this had increased to 72·5 %. Among children up to 12 years old, low- or no-sugar/diet drinks were the most commonly consumed beverages.

Correlations were observed between sociodemographic characteristics and beverage consumption (see online Supplemental Tables S1–S5). A greater proportion of households in the low-income group had children of any age who consumed low- or no-sugar/diet drinks (78·1 %), compared to middle-income (75·6 %) and high-income (66·3 %) consumers (*P* = 0·043).

A higher overall proportion of households with degree/higher education (83·3 %/85·3 %) consumed sugary drinks compared with those of high school (A Level/GCSE) education (80·2 %/80·6 %) and those with no/other education (74·6 %) (*P* = 0·034). However, in regard to just children’s consumption, a higher proportion of respondents with high school (A Level/GCSE) education (66·0 %/62·9 %) had children who drank sugary drinks compared with households with degree (51·3 %) and other higher education (57·8 %) and no/other education (47·3 %) (*P* = 0·026).

A higher proportion of households with younger (<40 years) respondents consumed low- or no-sugar/diet drinks (87·7 %) compared with those with respondents in the middle age group (40–49 years) (85·3 %) or older (50+ years) age group (76·1 %) (*P* = 0·012). A significantly greater proportion of households with younger respondents had children aged 13–18 years who consumed low- or no-sugar/diet drinks (76·3 %), compared with older respondents (55·0 %) (*P* = 0·012). This was also seen with children overall (76·1 % *v*. 60·0 %) (*P* = 0·001). However, a significantly greater proportion of households with older respondents had children aged 13–18 years who consumed sugary beverages (80·8 %), compared with younger respondents (55·3 %) (*P* < 0·001). This was also seen with children overall (73·6 % *v*. 49·7 %) (*P* < 0·001).

A higher proportion of households with a respondent with healthy BMI had children aged 7–12 years who consumed sugary drinks (61·8 %) compared with households where the main respondent was overweight (49·3 %) or obese (41·3 %) (*P* = 0·030).

Families of 4–5 people had the greatest low- or no-sugar/diet drinks consumption (87·4 %) compared with 76·1 % of small households (2–3 members) and 81·1 % of bigger households (6+ members) (*P* = 0·003). There were no significant associations found between intra-household self-reported consumption and SES or region.

### Adult–child consumption correlation

There was strong evidence to suggest that the odds of children consuming beverages were significantly increased when adults in the household consumed the same beverages (Table 2). The adult–child consumption correlation was strongest in older children. After adjusting for SES, region and age of respondent, children aged 13–18 years had over nine times the odds of consuming sugary drinks if adults in the household consumed sugary drinks (adjusted OR 9·55, (95 % CI 5·38, 17·00), *P* < 0·001), and eight times the odds of consuming low- or no-sugar/diet drinks if adults in the household consumed low- or no-sugar/diet drinks (adjusted OR 8·12, (95 % CI 4·71, 13·97), *P* < 0·001). The correlation was weaker for children aged 0–6 years and sugary drinks (adjusted OR 3·52, (95 % CI 1·34, 9·23), *P* = 0·010). The only group with which there was no evidence for correlation was in children aged 0–6 years and adult low- or no-sugar/diet drinks consumption (adjusted OR 1·63, (95 % CI 0·85, 3·12), *P* = 0·14). The adult–child consumption correlation effect was strongest for juice consumption in children overall (adjusted OR 6·39, (95 % CI 4·39, 9·32), *P* < 0·001). There was no evidence of effect modification of child–parent consumption patterns by sociodemographic groups (see online Supplemental Tables S6–S9).

**Table 2:** Crude and adjusted odds of children (of different ages) consuming beverages if adults consume beverages

Age category	Type of OR	*n*	Sugar			Low- or no-sugar/diet			Juice		
			OR	95 % CI	*P* value	OR	95 % CI	*P* value	OR	95 % CI	*P* value
All children under 18 years	Crude OR	603	4·48	3·05, 6·56	<0·001	4·54	3·12, 6·63	<0·001	6·14	4·25, 8·86	<0·001
	Full adjusted OR	603	5·23	3·47, 7·87	<0·001	4·75	3·20, 7·06	<0·001	6·39	4·39, 9·32	<0·001
Children 0–6 years	Crude OR	195	3·30	1·30, 8·36	0·006	1·77	0·95, 3·28	0·072	4·72	2·51, 8·85	<0·001
	Full adjusted OR	195	3·52	1·34, 9·23	0·010	1·63	0·85, 3·12	0·137	5·06	2·59, 9·91	<0·001
Children 7–12 years	Crude OR	312	5·85	3·25, 10·53	<0·001	3·56	2·05, 6·17	<0·001	7·38	4·39, 12·41	<0·001
	Full adjusted OR	312	5·75	3·16, 10·44	<0·001	3·79	2·13, 6·73	<0·001	7·80	4·52, 13·48	<0·001
Children 13+ years	Crude OR	334	7·98	4·75, 13·41	<0·001	7·51	4·50, 12·54	<0·001	6·18	3·80, 10·05	<0·001
	Full adjusted OR	334	9·55	5·38, 17·0	<0·001	8·12	4·71, 13·97	<0·001	6·28	3·83, 10·28	<0·001

Notes: *n* denotes number of households; fully adjusted models include categorical variables of socio-economic status, region, household size and age of respondent.

Highest qualification, income category and BMI category were excluded due to missing values for some respondents.

### Child–child consumption correlation

In households that had children in multiple age groups, having older siblings who consume beverages considerably increased the odds of younger children consuming these beverages too (see online Supplemental Table S10). There was strong evidence for increased odds of younger children consuming beverages if older children were consuming beverages (in the range of OR 11–121) across all age groups and beverages analysed, except for juice consumption of 0–6-year-olds who have a sibling of 13–18-year-olds who consumes. The data were too sparse across all categories to assess the impact of sociodemographic characteristics on child–child consumption.

## Discussion

The exploratory analysis undertaken in this study uncovered a number of intra-household non-alcoholic beverage consumption patterns in Great Britain. There was a clear association between increasing age and SSB consumption, with consumption doubling when children are 13–18 years old in comparison to those under 6 years old. This was in line with existing literature^(35,36)^. However, low- or no-sugar/diet drinks were the dominant beverage category with consistently high consumption across different age groups, including among the youngest. Accordingly, a greater proportion of households with older respondents reported consuming sugary drinks (reflecting the higher SSB consumption by older children in these households), and a greater proportion of households with younger respondents reported consuming low- or no-sugar/diet drinks (reflecting the higher consumption of low- or no-sugar/diet drinks by younger children in these households). Low- or no-sugar/diet drink consumption among young children is not surprising as concentrated drinks and juice drinks targeted for children in the UK market are often sweetened with non-caloric sweeteners^(37)^. While the prevalence of SSB consumption increases with the age of the child, there is no equivalent reduction in low- or no-sugar/diet drink consumption, suggesting that as the child ages, SSB may complement rather than substituting the existing consumption preferences.

A unique finding in this study was that overall self-reported sugary drink consumption was higher among those with tertiary education compared to those with only high school education or other/no education which contrasts with existing literature^(19,24,38,39)^. Similarly, a higher proportion of low-income households had children of any age who consumed low- or no-sugar/diet drinks, compared with middle- or high-income households. These findings could reflect a higher educated tendency to favour ‘natural’ sugar/sucrose-containing drinks rather than artificially sweetened beverages. It may also be that better educated adults are more aware of the divisive evidence surrounding the health impacts of artificial sweeteners; the health effects of which are inconclusive and remain an important evidence gap^(40)^. Indeed, in a poll of 2000 UK consumers, only 39 % of respondents believed artificial sweeteners were any healthier than sugar, with greater scepticism amongst young adult age groups^(41)^. This may also be linked to finding that those households where the respondent had healthy BMI reported a greater proportion of children aged 7–12 years consuming sugary drinks. This could imply that children are more likely to be allowed to consume sugary drinks when weight is not problematic. There could also be interplays with other non-communicable disease risk factors, for example, higher educated/wealthier families are likely to be more physically active^(42,43)^.

An increase in the consumption of pure fruit juice amongst UK children and adults has been one of the most important shifts in beverage consumption in the past decades^(44)^. In this study, pure fruit juice consumption was very stable across different age brackets, varying only from 51·8 % of households with children aged 0–6 consuming to 62·5 % of children aged 7–12 consuming, with adults and older children in between. In contrast with existing literature^(45)^, we did not find any association between household income and pure fruit juice consumption.

When we think about an adult purchaser, we must consider their consumption in the context of the interconnected home environment. Price signals are just one factor influencing purchase and may influence what is purchased for adults differently to what is purchased for children. Adult consumption is closely tied to the children’s consumption, and this study has demonstrated that these ties strengthen considerably as a child ages. By the time a child is in adolescence, they have over nine times the odds of consuming sugary drinks and eight times the odds of consuming low- or no-sugar/diet drinks, if adults in the household do so. While the correlation is strong, it is not possible to unequivocally determine the direction of causality, and there may be many factors whereby children influence the consumption of adults. For example, the increase in adult–child consumption correlation by teenagehood may reflect the greater autonomy that older children have in their own beverage consumption, and that they may develop preferences outside the home that subsequently influences adult consumption at home. Adolescents in the UK have the highest sugary drink consumption in Europe^(9,46)^, and adolescents have been shown generally to be more price-sensitive to beverages^(47)^ and other health-harming products^(48)^, yet also have established brand loyalty^(49)^. The child–adult consumption correlation found in this study was significantly stronger than other studies. Grimm *et al*.^(26)^ found that 8–13 years old American children had 2·88 times the odds (95 % CI 1·76, 4·72) of consuming soft drinks regularly if their parents did so. Ha *et al.* ^(22)^ looked exclusively at Australian infants aged up to 6–9 months and found that they had 1·6 times the odds (95 % CI 1·2, 2·3) of consuming sugary drinks if their mothers did so. Whilst our study had a bigger age range in the youngest group (0–6 years), the odds of these children consuming sugary drinks if adults in the household did were higher at 3·52 (95 % CI 1·34, 9·23, *P* = 0·010).

Findings in this study may suggest that families with younger children may be more sensitive to interventions to reduce SSB consumption, as they are more easily able to discriminate who in the family consumes what beverages. Such interventions may include fiscal policies such as SSB taxation, as well as tighter regulation of advertisement and promotion of SSB. In children up to the age of 6 years, parent–child correlations were the weakest, likely to be reflecting the greater control that adults have over a child’s food and drink consumption. They might thus also be more responsive to price signals as they feel less pressure from these young children who have not yet established autonomous consumption habits and preferences. However, this study also found sibling beverage consumption to be highly correlated, with younger children far more likely to consume beverages if they have older siblings that do. This may suggest that beverage restriction in children becomes much more challenging when there are older children in the household who can act as beverage consumption role models to younger children. Ultimately, the relative impact of sibling consumption as compared to adult consumption, as well as the direction of causality, is difficult to assess.

Interestingly, this study did not find any significant differences in child–parent consumption across sociodemographic groups. Considering beverage consumption patterns showed variability across sociodemographic groups, consistent with previous literature, this strong consumption correlation across all strata may suggest that limiting the consumption by children in the presence of consuming adults is difficult for all. The data were too sparse to assess the child–child consumption patterns across sociodemographic strata – this could be a focus of further research.

This study has revealed a dominance of low- or no-sugar/diet drink across different age groups and sociodemographic strata. This dominance was particularly driven by the youngest age group, where double the number of households had children aged 0–6 years consuming low- or no-sugar/diet drinks as compared to sugary drinks. Whether this is by parental choice or due to wider market shifts and reformulation towards low- or no-sugar/diet drinks for young children would require a deeper analysis. The absence of sugar in low- or no-sugar/diet beverages may contribute to low- or no-sugar/diet drinks being widely perceived as a ‘health neutral’ alternative, and one that is appropriate for all members of the family. This may well be influenced by stronger marketing and availability of low- or no-sugar/diet drink products, in light of increasing media and public health attention on the impacts of sugar on health.

### Limitations and strengths

One of the main limitations of this study was that consumption volumes or frequency amongst household members were not recorded, and therefore relative volumes of consumption amongst adults and children of different ages remain unknown. While the word ‘consumption’ may be interpreted differently by different respondents in terms of frequency or quantity, it nonetheless remains a reasonable indication of intra-household allocation in households that regularly purchase beverages. A related limitation is that we do not know the effect of reporting consumption by proxy (by the main shopper of the household) rather than individual self-reporting for each household member. The main shopper may over- or underestimate the relationship in consumption between household members. Regardless of these limitations, this study is, to the knowledge of the authors, the first attempt to estimate adult–child and child–child consumption patterns of non-alcoholic beverages in Great Britain and sheds important light into these relationships.

The inclusion criteria in the study of households who had purchased at least 2 l of non-alcoholic beverages every month in the 6 months preceding the survey, and who had at least one child under the age of 18 years, meant that the sample was not necessarily representative of the whole population. However, the sample was constructed as such to inform about preferences of the population generally targeted by childhood obesity policies.

Exploring consumption differences of beverages inside or outside of the home was not within the scope of this study, and therefore we cannot distinguish differences based on the data used. The social and economic factors influencing beverage consumption inside *v*. outside the home are complex and remain an important area of study.

While there were some patterns observed by BMI and income of the respondents, the missing data is an important limitation and should be considered with caution. Of the three-quarters of the sample that provided height and weight data, there was a lower prevalence of overweight and obesity compared to the general UK population (24·1 % *v*. 35 %, and 19 % *v*. 29 %, respectively). This may suggest that people who were overweight or obese were less likely to disclose their weight and height to Kantar. However, some differences could be due to sampling criteria applied. Given that the sociodemographic variables are collected by Kantar on an annual basis rather than through the survey in this study, it is unlikely however that the missingness of height and weight data was associated with self-reported consumption. Regardless, the sample size for these variables was smaller in the bivariate analyses and there may be some impact from this, particularly on statistical significance and therefore these findings should be considered descriptive.

Finally, this study was cross-sectional, meaning it is not possible to observe any changes in the patterns that may have occurred over time. The public awareness around the health impacts of SSB has grown in the last decade. As such, different cohorts of adults as well as their children represented in this study would have had different exposures to public health interventions and policies on this issue which could be driving the findings observed by the age of respondent. However, with lack of longitudinal data, we cannot formally test this. Additionally, in 2018, the UK Government introduced the Soft Drinks Industry Levy which has now been shown to have triggered a wide-scale reformulation effort by the industry to reduce sugar from SSB and thus have led to a decrease in sugar purchases from this source from 2016 when the levy was announced^(50)^. However, many of the reformulations reduced the sugar content below 5 g/100 ml to avoid the levy (rather than reduce it to close to 0 g) which in the context of this study would still be classified under sugary drinks.

### Policy implications

The strength and magnitude of the child–adult consumption association found in this study suggest that strategies to reduce child consumption of sweetened beverages need to begin very early in life, when child food development preferences may be most modifiable^(21)^. Children are very likely to adopt their parent’s consumption patterns (and those of older siblings) no matter what their sociodemographic background. While adolescence may be an important intervention point, the findings here suggest that children are demonstrating beverage-drinking consumption patterns before they turn 13 years of age.

It is clear that many Great Britain households are reporting consumption of low- or no-sugar/diet drinks, with consumption occurring at all ages. This is likely having a good impact on total free sugar intake. However, it reveals an urgent need for more research on the health impacts of non-caloric sweeteners, which still remains inconclusive^(40)^. This is especially pressing for young children who appear to be consuming these beverages more than SSB.

## Footnotes

1 Respondents were familiar with the beverage choices based on the choice experiment they had completed prior to answering the question about consumption by household members. Further information about the choice experiment and how beverages were displayed can be found in Cornelsen *et al.* (2020).

2 AB – higher and intermediate managerial, administrative, professional occupations, C1 – supervisory, clerical and junior managerial, C2 – skilled manual occupations, D – semi-skilled and unskilled manual occupations, E – unemployed and lowest grade occupations.

3 Three categories of: £0–19 999, £20 000–39 999 and £40 000 and above.

4 Five categories of: London/Anglia, South/South West, North/Yorkshire/Lancashire, Midlands/Wales/West, Scotland.

5 Five categories of: degree or higher; higher education; high school (A level); high school (GCSE); other/none.

6 These figures are reported by households annually together with remaining sociodemographic characteristics. BMI was calculated using the formula BMI = (weight in kilograms)/(height in metres)^2.

## Supplementary material

For supplementary material accompanying this paper visit https://doi.org/10.1017/S1368980021005061. click here to view supplementary material
