# Supplementary material for: Like parent, like child: a cross-sectional study of intra-household consumption patterns of non-alcoholic beverages among British households with children
Source: Public Health Nutr. 2021 Dec 27;25(7):1771–9. doi: 10.1017/S1368980021005061 (PMC9991679; doi:10.1017/S1368980021005061)
Supplement: Supplementary file 1 [file S1368980021005061sup001.docx]

# Supplementary tables

**Table S1**. Proportion of households where at least one person consumes beverage *n = 603*

| **Variable** | **Category** | **Total number of households** | **Number (%) of households where at least one person consumes beverage** | | | | | |
| --- | --- | --- | --- | --- | --- | --- | --- | --- |
|  |  |  | **Sugary drinks** | | **Low or no-sugar/diet drinks** | | **Juice** | |
| **Socio-economic status of respondent** | High (AB) | 102 | 87 (85.3%) | | 81 (79.4%) | | 82 (80.4%) | |
|  | Middle (C1, C2) | 348 | 281 (80.8%) | | 294 (84.5%) | | 262 (75.3%) | |
|  | Low (DE) | 153 | 122 (79.7%) | | 129 (84.3%) | | 107 (69.9%) | |
|  | *χ^2^ (df)* | | | 1.38 (2) | | 1.56 (2) | | 3.66 (2) |
|  | *p value* | | | 0.50 | | 0.46 | | 0.16 |
| **Household income group^a^ (£)** | 0-19,999 | 114 | 89 (78.1%) | | 100 (87.7%) | | 84 (73.7%) | |
|  | 20,000-39,999 | 225 | 186 (82.7%) | | 194 (86.2%) | | 168 (74.7%) | |
|  | 40,000+ | 178 | 147 (82.6%) | | 142 (79.8%) | | 144 (80.9%) | |
|  |  |  |  | |  | |  | |
|  | *χ^2^(df)* | | | 1.23 (2) | | 4.39 (2) | | 2.84 (2) |
|  | *p value* | | | 0.54 | | 0.11 | | 0.24 |
| **Region** | London/Anglia | 136 | 118 (86.8%) | | 108 (79.4%) | | 103 (75.7%) | |
|  | South/South West | 85 | 68 (80.0%) | | 70 (82.4%) | | 68 (80.0%) | |
|  | North/Yorkshire/  Lancashire | 177 | 138 (77.9%) | | 153 (86.4%) | | 127 (71.8%) | |
|  | Midlands/Wales and West | 159 | 129 (81.1%) | | 137 (86.2%) | | 117 (73.6%) | |
|  | Scotland | 46 | 37 (80.4%) | | 36 (78.3%) | | 36 (78.3%) | |
|  | *χ^2^(df)* | | | 4.08 (4) | | 4.59 (4) | | 2.57 (4) |
|  | *p value* | | | 0.40 | | 0.33 | | 0.63 |
| **Highest qualification of respondent^b^** | Degree or higher | 156 | 130 (83.3%) | | 122 (78.2%) | | 125 (80.1%) | |
|  | Higher education | 109 | 93 (85.3%) | | 96 (88.1%) | | 85 (78.0%) | |
|  | High school (A Level) | 106 | 85 (80.2%) | | 88 (83.0%) | | 78 (73.6%) | |
|  | High school (GCSE) | 175 | 141 (80.6%) | | 151 (86.3%) | | 118 (67.4%) | |
|  | Other/none | 55 | 41 (74.6%) | | 45 (81.8%) | | 44 (80.0%) | |
|  | *χ^2^(df)* | | | 12.06 (5) | | 6.36 (5) | | 9.50 (5) |
|  | *p value* | | | 0.034 | | 0.27 | | 0.091 |
| **Age of respondent** | <40 years | 163 | 136 (83.4%) | | 143 (87.7%) | | 122 (74.9%) | |
|  | 40-49 years | 285 | 223 (78.3%) | | 243 (85.3%) | | 215 (75.4%) | |
|  | 50+ years | 155 | 131 (84.5%) | | 118 (76.1%) | | 114 (73.6%) | |
|  | *χ^2^(df)* | | | 3.29 (2) | | 8.91 (2) | | 0.19 (2) |
|  | *p value* | | | 0.19 | | 0.012 | | 0.91 |
| **BMI of respondent^c^** | BMI <25 | 193 | 166 (86.0%) | | 155 (80.3%) | | 150 (77.7%) | |
|  | Overweight 25-29.9 | 145 | 115 (79.3%) | | 124 (85.5%) | | 115 (79.3%) | |
|  | Obese 30+ | 117 | 95 (81.2%) | | 104 (88.9%) | | 83 (70.9%) | |
|  | *χ^2^(df)* | | | 2.81 (2) | | 4.31 (2) | | 2.81 (2) |
|  | *p value* | | | 0.24 | | 0.12 | | 0.25 |
| **Household size** | 2-3 people | 176 | 136 (77.3%) | | 134 (76.1%) | | 136 (77.3%) | |
|  | 4-5 people | 374 | 308 (82.4%) | | 327 (87.4%) | | 275 (73.5%) | |
|  | 6+ people | 53 | 46 (86.8%) | | 43 (81.1%) | | 40 (75.5%) | |
|  | *χ^2^(df)* | | | 3.20 (2) | | 11.38 (2) | | 0.90 (2) |
|  | *p value* | | | 0.20 | | 0.003 | | 0.64 |

Notes: ^a^ missing data for 86 respondents; ^b^missing data for 2 respondents; ^c^missing data for 148 respondents

**Table S2.** Proportion of households with children 0-6 years who have children 0-6 years that consume, *n = 195*

| **Variable** | **Category** | **Number of households with children aged 0-6** | **Number (%) of households with children 0-6 years who have children 0-6 years that consume** | | |
| --- | --- | --- | --- | --- | --- |
|  |  |  | **Sugary drinks** | **Low or no-sugar/diet drinks** | **Juice** |
| **Socio-economic status of respondent** | High (AB) | 34 | 13 (38.2%) | 19 (55.9%) | 22 (64.7%) |
|  | Middle (C1, C2) | 119 | 26 (30.3%) | 82 (68.9%) | 59 (49.6%) |
|  | Low (DE) | 42 | 17 (40.5%) | 27 (64.3%) | 20 (47.6%) |
|  | *χ^2^ (df)* | | 1.80 (2) | 2.03 (2) | 2.80 (2) |
|  | *p value* | | 0.41 | 0.36 | 0.25 |
| **Household income group (£)^a^** | 0-19,999 | 34 | 13 (28.2%) | 22 (64.7%) | 14 (41.2%) |
|  | 20,000-39,999 | 85 | 31 (36.5%) | 60 (70.6%) | 48 (56.5%) |
|  | 40,000+ | 49 | 14 (28.6%) | 31 (63.3%) | 24 (49.0%) |
|  |  |  |  |  |  |
|  | *χ^2^ (df)* | | 1.12 (2) | 0.883 (2) | 2.41 (2) |
|  | *p value* | | 0.57 | 0.64 | 0.30 |
| **Region** | London/Anglia | 40 | 16 (40.0%) | 24 (60.0%) | 20 (50.0%) |
|  | South/South West | 27 | 7 (25.9%) | 17 (63.0%) | 13 (48.2%) |
|  | North/Yorkshire/  Lancashire | 60 | 19 (31.7%) | 42 (70.0%) | 31 (51.7%) |
|  | Midlands/Wales and West | 59 | 23 (39.0%) | 39 (66.1%) | 33 (55.9%) |
|  | Scotland | 9 | 1 (11.1%) | 6 (66.7%) | 4 (44.4%) |
|  | *χ^2^ (df)* | | 4.33 (4) | 1.17 (4) | 0.80 (4) |
|  | *p value* | | 0.36 | 0.88 | 0.94 |
| **Highest qualification of respondent^b^** | Degree or higher | 54 | 17 (31.5%) | 34 (63.0%) | 35 (64.8%) |
|  | Higher education | 36 | 10 (27.8%) | 28 (77.8%) | 16 (44.4%) |
|  | High school (A Level) | 46 | 18 (39.1%) | 29 (63.0%) | 22 (47.8%) |
|  | High school (GCSE) | 40 | 13 (32.5%) | 27 (67.5%) | 16 (40.0%) |
|  | Other/none | 17 | 8 (47.1%) | 9 (52.9%) | 12 (70.6%) |
|  | *χ^2^ (df)* | | 3.68 (5) | 4.15 (5) | 11.52 (5) |
|  | *p value* | | 0.60 | 0.53 | 0.042 |
| **Age of respondent** | <40 years | 117 | 39 (33.3%) | 81 (69.2%) | 62 (53.0%) |
|  | 40-49 years | 61 | 17 (27.9%) | 38 (62.3%) | 29 (47.5%) |
|  | 50+ years | 17 | 10 (58.8%) | 9 (52.9%) | 10 (58.8%) |
|  | *χ^2^ (df)* | | 5.72 (2) | 2.19 (2) | 0.85 (2) |
|  | *p value* | | 0.057 | 0.34 | 0.66 |
| **BMI of respondent^c^** | BMI <25 | 70 | 27 (38.6%) | 53 (75.7%) | 37 (52.9%) |
|  | Overweight 25-29.9 | 43 | 14 (32.6%) | 29 (67.4%) | 25 (58.1%) |
|  | Obese 30+ | 38 | 14 (36.8%) | 21 (55.3%) | 23 (60.5%) |
|  | *χ^2^ (df)* | | 0.42 (2) | 4.77 (2) | 0.67 (2) |
|  | *p value* | | 0.81 | 0.092 | 0.72 |
| **Household size** | 2-3 people | 39 | 14 (35.9%) | 23 (59.0%) | 20 (51.3%) |
|  | 4-5 people | 127 | 41 (32.3%) | 91 (71.7%) | 68 (53.5%) |
|  | 6+ people | 29 | 11 (37.9%) | 14 (48.3%) | 13 (44.8%) |
|  | *χ^2^ (df)* | | 0.43 (2) | 6.68 (2) | 0.72 (2) |
|  | *p value* | | 0.81 | 0.035 | 0.70 |

Notes: ^a^ missing data for 27 respondents; ^b^missing data for 2 respondents; ^c^missing data for 44 respondents

**Table S3**. Proportion of households with children 7-12 years who have children 7-12 years that consume, *n = 312*

| **Variable** | **Category** | **Number of households with children aged 7-12** | **Number (%) of households with children 7-12 who have children 7-12 that consume** | | | | | |  |
| --- | --- | --- | --- | --- | --- | --- | --- | --- | --- |
|  |  |  | **Sugary drinks** | | **Low or no-sugar/diet drinks** | | **Juice** | |  |
| **Socio-economic status of respondent** | High (AB) | 53 | 26 (49.1%) | | 36 (67.9%) | | 31 (58.5%) | |  |
|  | Middle (C1, C2) | 184 | 98 (53.3%) | | 144 (78.3%) | | 121 (65.8%) | |  |
|  | Low (DE) | 75 | 40 (53.3%) | | 62 (82.7%) | | 43 (57.3%) | |  |
|  | *χ^2^ (df)* | | | 0.32 (2) | | 4.00 (2) | | 2.05 (2) | |
|  | *p value* | | | 0.85 | | 0.14 | | 0.36 | |
| **Household income group (£)^a^** | 0-19,999 | 57 | 32 (56.1%) | | 47 (82.5%) | | 34 (59.7%) | |  |
|  | 20,000-39,999 | 119 | 56 (47.1%) | | 95 (79.8%) | | 73 (61.3%) | |  |
|  | 40,000+ | 92 | 50 (54.4%) | | 68 (73.9%) | | 62 (67.4%) | |  |
|  |  |  |  | |  | |  | |  |
|  | *χ^2^ (df)* | | | 1.73 (2) | | 1.79 (2) | | 1.18 (2) | |
|  | *p value* | | | 0.42 | | 0.41 | | 0. 56 | |
| **Region** | London/Anglia | 69 | 38 (55.1%) | | 53 (76.8%) | | 50 (72.5%) | |  |
|  | South/South West | 39 | 21 (53.9%) | | 31 (79.5%) | | 24 (61.5%) | |  |
|  | North/Yorkshire/  Lancashire | 95 | 45 (47.4%) | | 74 (77.9%) | | 58 (61.1%) | |  |
|  | Midlands/Wales and West | 85 | 48 (56.5%) | | 65 (76.5%) | | 44 (51.8%) | |  |
|  | Scotland | 24 | 12 (50.0%) | | 19 (79.2%) | | 19 (79.2%) | |  |
|  | *χ^2^ (df)* | | | 1.81 (4) | | 0.21 (4) | | 10.05 (4) | |
|  | *p value* | | | 0.77 | | 0.96 | | 0.040 | |
| **Highest qualification of respondent^b^** | Degree or higher | 74 | 37 (50.0%) | | 55 (74.3%) | | 51 (68.9%) | |  |
|  | Higher education | 64 | 36 (56.3%) | | 50 (78.1%) | | 44 (68.8%) | |  |
|  | High school (A Level) | 57 | 35 (61.4%) | | 40 (70.2%) | | 34 (59.7%) | |  |
|  | High school (GCSE) | 90 | 47 (52.2%) | | 78 (86.7%) | | 49 (54.4%) | |  |
|  | Other/none | 26 | 9 (34.6%) | | 18 (69.2%) | | 16 (61.5%) | |  |
|  | *χ^2^ (df)* | | | 6.80 (5) | | 7.86 (5) | | 5.67 (5) | |
|  | *p value* | | | 0.236 | | 0.16 | | 0.34 | |
| **Age of respondent** | <40 years | 97 | 52 (53.6%) | | 72 (74.2%) | | 63 (65.0%) | |  |
|  | 40-49 years | 173 | 91 (52.9%) | | 135 (78.0%) | | 108 (62.4%) | |  |
|  | 50+ years | 42 | 21 (50.0%) | | 35 (83.3%) | | 24 (57.1%) | |  |
|  | *χ^2^ (df)* | | | 0.15 (2) | | 1.45 (2) | | 0.76 (2) | |
|  | *p value* | | | 0.93 | | 0.49 | | 0.68 | |
| **BMI of respondent^c^** | BMI <25 | 102 | 63 (61.8%) | | 82 (80.4%) | | 64 (62.8%) | |  |
|  | Overweight 25-29.9 | 75 | 37 (49.3%) | | 58 (77.3%) | | 49 (65.3%) | |  |
|  | Obese 30+ | 63 | 26 (41.3%) | | 45 (71.4%) | | 41 (65.1%) | |  |
|  | *χ^2^ (df)* | | | 7.00 (2) | | 1.78 (2) | | 0.16 (2) | |
|  | *p value* | | | 0.030 | | 0.41 | | 0.93 | |
| **Household size** | 2-3 people | 50 | 20 (40.0%) | | 40 (80.0%) | | 29 (58.0%) | |  |
|  | 4-5 people | 223 | 121 (54.3%) | | 177 (79.4%) | | 140 (62.8%) | |  |
|  | 6+ people | 39 | 23 (59.0%) | | 25 (64.1%) | | 26 (66.7%) | |  |
|  | *χ^2^ (df)* | | | 4.07 (2) | | 4.65 (2) | | 0.73 (2) | |
|  | *p value* | | | 0.13 | | 0.098 | | 0.70 | |

Notes: ^a^ missing data for 44 respondents; ^b^ missing data for 1 respondent; ^c^missing data for 72 respondents

**Table S4.** Proportion of households with children 13-18 years who have children 13-18 years that consume, *n = 334*

| **Variable** | **Category** | **Number of households with children aged 13-18** | **Number (%) of households with children 13-18 who have children 13-18 that consume** | | |
| --- | --- | --- | --- | --- | --- |
|  |  |  | **Sugary drinks** | **Low or no-sugar/diet drinks** | **Juice** |
| **Socio-economic status of respondent** | High (AB) | 57 | 39 (68.4%) | 34 (59.7%) | 36 (63.2%) |
|  | Middle (C1, C2) | 175 | 120 (68.6%) | 115 (65.7%) | 108 (62.7%) |
|  | Low (DE) | 102 | 67 (65.7%) | 68 (66.7%) | 60 (58.8%) |
|  | *χ^2^ (df)* | | 0.26 (2) | 0.88 (2) | 0.35 (2) |
|  | *p value* | | 0.88 | 0.64 | 0.84 |
| **Household income group (£)^a^** | 0-19,999 | 69 | 42 (60.9%) | 49 (71.0%) | 41 (59.4%) |
|  | 20,000-39,999 | 123 | 92 (74.8%) | 83 (67.5%) | 77 (62.6%) |
|  | 40,000+ | 93 | 60 (64.5%) | 57 (61.3%) | 63 (67.7%) |
|  |  |  |  |  |  |
|  | *χ^2^ (df)* | | 4.75 (2) | 1.81 (2) | 1.26 (2) |
|  | *p value* | | 0.09 | 0.405 | 0.53 |
| **Region** | London/Anglia | 74 | 54 (73.0%) | 46 (62.2%) | 48 (34.9%) |
|  | South/South West | 52 | 31 (59.6%) | 34 (65.4%) | 33 (63.5%) |
|  | North/Yorkshire/  Lancashire | 102 | 66 (64.7%) | 68 (66.7%) | 59 (57.8%) |
|  | Midlands/Wales and West | 83 | 60 (72.3%) | 54 (65.1%) | 51 (61.5%) |
|  | Scotland | 23 | 15 (65.2%) | 15 (65.2%) | 13 (56.5%) |
|  | *χ^2^ (df)* | | 3.78 (4) | 0.39 (4) | 1.23 (4) |
|  | *p value* | | 0.44 | 0.98 | 0.87 |
| **Highest qualification of respondent** | Degree or higher | 74 | 46 (62.2%) | 42 (56.8%) | 47 (63.5%) |
|  | Higher education | 55 | 39 (70.9%) | 36 (65.5%) | 36 (65.5%) |
|  | High school (A Level) | 57 | 39 (68.4%) | 38 (66.7%) | 33 (57.9%) |
|  | High school (GCSE) | 115 | 83 (72.2%) | 81 (70.4%) | 67 (58.3%) |
|  | Other/none | 33 | 19 (57.6%) | 20 (60.6%) | 21 (63.6%) |
|  | *χ^2^ (df)* | | 3.91 (4) | 4.06 (4) | 1.35 (4) |
|  | *p value* | | 0.42 | 0.40 | 0.85 |
| **Age of respondent** | <40 years | 38 | 21 (55.3%) | 29 (76.3%) | 20 (52.6%) |
|  | 40-49 years | 176 | 108 (61.4%) | 122 (69.3%) | 110 (62.5%) |
|  | 50+ years | 120 | 97 (80.8%) | 66 (55.0%) | 74 (61.7%) |
|  | *χ^2^ (df)* | | 15.38 (2) | 8.85 (2) | 1.31 (2) |
|  | *p value* | | <0.001 | 0.012 | 0.52 |
| **BMI of respondent^b^** | BMI <25 | 106 | 76 (71.7%) | 66 (62.3%) | 68 (64.2%) |
|  | Overweight 25-29.9 | 77 | 51 (66.2%) | 54 (70.1%) | 47 (61.0%) |
|  | Obese 30+ | 63 | 44 (69.8%) | 42 (66.7%) | 36 (57.1%) |
|  | *χ^2^ (df)* | | 0.63 (2) | 1.25 (2) | 0.82 (2) |
|  | *p value* | | 0.73 | 0.54 | 0.66 |
| **Household size** | 2-3 people | 97 | 59 (60.8%) | 53 (54.6%) | 63 (65.0%) |
|  | 4-5 people | 202 | 143 (70.8%) | 142 (70.3%) | 119 (58.9%) |
|  | 6+ people | 35 | 24 (68.6%) | 22 (62.9%) | 22 (62.9%) |
|  | *χ^2^ (df)* | | 2.99 (2) | 7.14 (2) | 1.06 (2) |
|  | *p value* | | 0.22 | 0.028 | 0.59 |

Notes: ^a^ missing data for 49 respondents; ^b^missing data for 88 respondents

**Table S5**. Proportion of households where any child (aged 0-18 years) consumes, *n = 603*

| **Variable** | **Category** | **Number of households with children of any age** | **Number (%) of households with children of any age who have children of any age that consume** | | |
| --- | --- | --- | --- | --- | --- |
|  |  |  | **Sugary drinks** | **Low or no-sugar/diet drinks** | **Juice** |
| **Socio-economic status of respondent** | High (AB) | 102 | 58 (56.9%) | 66 (64.7%) | 68 (66.7%) |
|  | Middle (C1, C2) | 348 | 198 (56.9%) | 256 (73.6%) | 226 (64.9%) |
|  | Low (DE) | 153 | 93 (60.8%) | 112 (73.2%) | 93 (60.8%) |
|  | *χ^2^ (df)* | | 0.71 (2) | 3.22 (2) | 1.13 (2) |
|  | *p value* | | 0.70 | 0.20 | 0.57 |
| **Household income group (£)^a^** | 0-19,999 | 114 | 66 (57.9%) | 89 (78.1%) | 71 (62.3%) |
|  | 20,000-39,999 | 225 | 138 (61.3%) | 170 (75.6%) | 150 (66.7%) |
|  | 40,000+ | 178 | 96 (53.9%) | 118 (66.3%) | 123 (69.1%) |
|  |  |  |  |  |  |
|  | *χ^2^ (df)* | | 2.24 (2) | 6.28 (2) | 1.45 (2) |
|  | *p value* | | 0.33 | 0.043 | 0.48 |
| **Region** | London/Anglia | 136 | 84 (61.8%) | 93 (68.4%) | 92 (67.7%) |
|  | South/South West | 85 | 46 (54.1%) | 61 (71.8%) | 58 (68.2%) |
|  | North/Yorkshire/  Lancashire | 177 | 100 (56.5%) | 135 (76.3%) | 111 (62.7%) |
|  | Midlands/Wales and West | 159 | 95 (59.8%) | 112 (70.4%) | 96 (60.4%) |
|  | Scotland | 46 | 24 (52.2%) | 33 (71.7%) | 30 (65.2%) |
|  | *χ^2^ (df)* | | 2.32 (4) | 2.68 (4) | 2.51 (4) |
|  | *p value* | | 0.68 | 0.61 | 0.64 |
| **Highest qualification of respondent^b^** | Degree or higher | 156 | 80 (51.3%) | 103 (66.0%) | 107 (68.6%) |
|  | Higher education | 109 | 63 (57.8%) | 85 (78.0%) | 74 (67.9%) |
|  | High school (A Level) | 106 | 70 (66.0%) | 74 (69.8%) | 66 (62.3%) |
|  | High school (GCSE) | 175 | 110 (62.9%) | 135 (77.1%) | 102 (58.3%) |
|  | Other/none | 55 | 26 (47.3%) | 35 (63.6%) | 37 (67.3%) |
|  | *χ^2^ (df)* | | 12.74 (5) | 9.92 (5) | 5.19 (5) |
|  | *p value* | | 0.026 | 0.077 | 0.39 |
| **Age of respondent** | <40 years | 163 | 81 (49.7%) | 124 (76.1%) | 102 (62.6%) |
|  | 40-49 years | 285 | 154 (54.0%) | 217 (76.1%) | 188 (66.0%) |
|  | 50+ years | 155 | 114 (73.6%) | 93 (60.0%) | 97 (62.6%) |
|  | *χ^2^ (df)* | | 21.82 (2) | 14.83 (2) | 0.75 (2) |
|  | *p value* | | <0.001 | 0.001 | 0.69 |
| **BMI of respondent^c^** | BMI <25 | 193 | 124 (64.3%) | 140 (72.5%) | 131 (67.9%) |
|  | Overweight 25-29.9 | 145 | 83 (57.2%) | 109 (75.2%) | 99 (68.3%) |
|  | Obese 30+ | 117 | 62 (53.0%) | 81 (69.2%) | 75 (64.1%) |
|  | *χ^2^ (df)* | | 4.13 (2) | 1.15 (2) | 0.62 (2) |
|  | *p value* | | 0.13 | 0.56 | 0.73 |
| **Household size** | 2-3 people | 176 | 89 (50.6%) | 109 (61.9%) | 109 (61.9%) |
|  | 4-5 people | 374 | 225 (60.2%) | 290 (77.5%) | 243 (65.0%) |
|  | 6+ people | 53 | 35 (66.0%) | 35 (66.0%) | 35 (66.0%) |
|  | *χ^2^ (df)* | | 6.10 (2) | 15.47 (2) | 0.57 (2) |
|  | *p value* | | 0.047 | <0.001 | 0.75 |

Notes: ^a^ missing data for 86 respondents, ^b^ missing data for 2 respondents; ^c^ missing data for 148 respondents

**Table S6.** Tests for effect modification of socio-demographic variables on child-adult consumption correlation in all children under 18 years of age.

|  | **Sugary drinks** | | | | | **Low or no-sugar/diet drinks** | | | | | **Juice** | | | | |
| --- | --- | --- | --- | --- | --- | --- | --- | --- | --- | --- | --- | --- | --- | --- | --- |
|  | **Mantel-Haenszel** | | **Test for homogeneity of ORs** | | | **Mantel-Haenszel** | | **Test for homogeneity of ORs** | | | **Mantel-Haenszel** | | **Test for homogeneity of ORs** | | |
|  | **Adjusted OR** | **95% CI** | **χ^2^** | **df** | **p value** | **Adjusted OR** | **95% CI** | **χ^2^** | **df** | **p value** | **Adjusted OR** | **95% CI** | **χ^2^** | **df** | **p value** |
| **SES of respondent** | 4.48 | 2.99, 6.72 | 2.84 | 2 | 0.24 | 4.45 | 2.99, 6.64 | 1.34 | 2 | 0.51 | 6.16 | 4.10, 9.23 | 0.74 | 2 | 0.69 |
| **Household income group (£)^a^** | 4.18 | 2.71, 6.46 | 0.10 | 2 | 0.95 | 4.23 | 2.74, 6.54 | 1.09 | 2 | 0.58 | 5.84 | 3.76, 9.08 | 0.10 | 2 | 0.95 |
| **Region** | 4.43 | 2.96, 6.65 | 2.82 | 4 | 0.59 | 4.50 | 3.01, 6.73 | 3.14 | 4 | 0.53 | 6.03 | 4.04, 9.00 | 4.18 | 4 | 0.38 |
| **Highest qualification of respondent^b^** | 4.96 | 3.23, 7.59 | 6.52 | 4 | 0.16 | 4.56 | 3.04, 6.84 | 2.2 | 4 | 0.70 | 6.01 | 4.01, 9.02 | 1.71 | 4 | 0.79 |
| **Age of respondent** | 5.13 | 3.34, 7.89 | 0.19 | 2 | 0.91 | 4.81 | 3.19, 7.27 | 2.83 | 2 | 0.24 | 6.20 | 4.13, 9.30 | 0.090 | 2 | 0.96 |
| **BMI of respondent^c^** | 4.12 | 2.58, 6.57 | 1.32 | 2 | 0.52 | 4.49 | 2.79, 7.22 | 2.22 | 2 | 0.33 | 6.25 | 3.88, 10.06 | 1.63 | 2 | 0.44 |
| **Household size** | 4.40 | 2.93, 6.60 | 0.26 | 2 | 0.88 | 4.52 | 3.01, 6.79 | 0.87 | 2 | 0.65 | 6.21 | 4.14, 9.30 | 2.94 | 2 | 0.23 |

Notes: ^a^ missing data for 86 respondents; ^b^ missing data for 2 respondents; ^c^ missing data for 148 respondents

**Table S7**. Tests for effect modification of socio-demographic variables on child-adult consumption correlation in children aged 0-6 years.

|  | **Sugary drinks** | | | | | **Low or no-sugar/diet drinks** | | | | | **Juice** | | | | |
| --- | --- | --- | --- | --- | --- | --- | --- | --- | --- | --- | --- | --- | --- | --- | --- |
|  | **Mantel-Haenszel** | | **Test for homogeneity of ORs** | | | **Mantel-Haenszel** | | **Test for homogeneity of ORs** | | | **Mantel-Haenszel** | | **Test for homogeneity of ORs** | | |
|  | **Adjusted OR** | **95% CI** | **χ^2^** | **df** | **p value** | **Adjusted OR** | **95% CI** | **χ^2^** | **df** | **p value** | **Adjusted OR** | **95% CI** | **χ^2^** | **df** | **p value** |
| **SES of respondent** | 3.39 | 1.29, 8.87 | 0.03 | 2 | 0.99 | 1.78 | 0.95, 3.33 | 1.52 | 2 | 0.47 | 4.50 | 2.27, 8.93 | 2.06 | 2 | 0.36 |
| **Household income group (£)^a^** | 3.38 | 1.22, 9.38 | 1.44 | 2 | 0.49 | 1.58 | 0.82, 3.02 | 5.7 | 2 | 0.06 | 4.56 | 2.20, 9.42 | 2.31 | 2 | 0.31 |
| **Region** | 3.54 | 1.32, 9.46 | 2.48 | 4 | 0.65 | 1.73 | 0.92, 3.25 | 2.26 | 4 | 0.69 | 4.62 | 2.39, 8.93 | 8.66 | 4 | 0.07 |
| **Highest qualification of respondent^b^** | 3.69 | 1.30, 10.46 | 2.59 | 4 | 0.63 | 2.04 | 1.07, 3.87 | 3.42 | 4 | 0.49 | 3.98 | 1.97, 8.03 | 0.52 | 4 | 0.97 |
| **Age of respondent** | 3.20 | 1.21, 8.44 | 0.35 | 2 | 0.84 | 1.75 | 0.93, 3.29 | 0.43 | 2 | 0.81 | 4.67 | 2.37, 9.23 | 0.23 | 2 | 0.89 |
| **BMI of respondent^c^** | 2.35 | 0.86, 6.47 | 3.56 | 2 | 0.17 | 1.86 | 0.87, 3.96 | 0.31 | 2 | 0.86 | 4.33 | 2.05, 9.14 | 5.84 | 2 | 0.05 |
| **Household size** | 3.29 | 1.26, 8.54 | 1.40 | 2 | 0.50 | 1.66 | 0.88, 3.13 | 0.48 | 2 | 0.79 | 5.06 | 2.51, 10.18 | 0.55 | 2 | 0.78 |

Notes: ^a^ missing data for 27 respondents; ^b^missing data for 2 respondents; ^c^missing data for 44 respondents

**Table S8**. Tests for effect modification of socio-demographic variables on child-adult consumption correlation in children aged 7-12 years.

|  | **Sugary drinks** | | | | | **Low or no-sugar/diet drinks** | | | | | **Juice** | | | | |
| --- | --- | --- | --- | --- | --- | --- | --- | --- | --- | --- | --- | --- | --- | --- | --- |
|  | **Mantel-Haenszel** | | **Test for homogeneity of ORs** | | | **Mantel-Haenszel** | | **Test for homogeneity of ORs** | | | **Mantel-Haenszel** | | **Test for homogeneity of ORs** | | |
|  | **Adjusted OR** | **95% CI** | **χ^2^** | **df** | **p value** | **Adjusted OR** | **95% CI** | **χ^2^** | **df** | **p value** | **Adjusted OR** | **95% CI** | **χ^2^** | **df** | **p value** |
| **SES of respondent** | 5.84 | 3.11, 11.0 | 0.07 | 2 | 0.97 | 3.50 | 1.97, 6.20 | 2.39 | 2 | 0.30 | 7.60 | 4.20, 13.77 | 0.52 | 2 | 0.77 |
| **Household income group (£)^a^** | 6.55 | 3.24, 13.22 | 0.14 | 2 | 0.93 | 3.53 | 1.86, 6.70 | 5.65 | 2 | 0.06 | 7.36 | 3.90, 13.89 | 0.69 | 2 | 0.71 |
| **Region** | 5.73 | 3.05, 10.76 | 2.33 | 4 | 0.67 | 3.62 | 2.03, 6.46 | 3.25 | 4 | 0.52 | 7.58 | 4.16, 13.83 | 0.44 | 4 | 0.98 |
| **Highest qualification of respondent^b^** | 6.32 | 3.25, 12.26 | 4.37 | 4 | 0.36 | 3.50 | 1.96, 6.27 | 1.59 | 4 | 0.81 | 7.22 | 4.03, 12.94 | 3.42 | 4 | 0.49 |
| **Age of respondent** | 5.85 | 3.11, 11.02 | 0.12 | 2 | 0.94 | 3.50 | 1.98, 6.22 | 0.54 | 2 | 0.76 | 7.30 | 4.08, 13.06 | 0.66 | 2 | 0.72 |
| **BMI of respondent^c^** | 7.07 | 3.27, 15.28 | 1.32 | 2 | 0.52 | 3.91 | 1.98, 7.70 | 0.53 | 2 | 0.77 | 7.48 | 3.83, 14.62 | 1.03 | 2 | 0.60 |
| **Household size** | 5.50 | 2.98, 10.15 | 4.30 | 2 | 0.12 | 3.72 | 2.07, 6.67 | 0.60 | 2 | 0.74 | 7.08 | 4.00, 12.51 | 3.70 | 2 | 0.16 |

Notes: ^a^ missing data for 44 respondents; ^b^ missing data for 1 respondent; ^c^ missing data for 72 respondents

**Table S9.** Tests for effect modification of socio-demographic variables on child-adult consumption correlation in children aged 13-18 years.

|  | **Sugary drinks** | | | | | **Low or no-sugar/diet drinks** | | | | | **Juice** | | | | |
| --- | --- | --- | --- | --- | --- | --- | --- | --- | --- | --- | --- | --- | --- | --- | --- |
|  | **Mantel-Haenszel** | | **Test for homogeneity of ORs** | | | **Mantel-Haenszel** | | **Test for homogeneity of ORs** | | | **Mantel-Haenszel** | | **Test for homogeneity of ORs** | | |
|  | **Adjusted OR** | **95% CI** | **χ^2^** | **df** | **p value** | **Adjusted OR** | **95% CI** | **χ^2^** | **df** | **p value** | **Adjusted OR** | **95% CI** | **χ^2^** | **df** | **p value** |
| **SES of respondent** | 7.93 | 4.43, 14.17 | 1.34 | 2 | 0.51 | 7.43 | 4.18, 13.21 | 0.65 | 2 | 0.72 | 6.14 | 3.60, 10.46 | 1.91 | 2 | 0.39 |
| **Household income group (£)^a^** | 7.70 | 4.08, 14.53 | 0.61 | 2 | 0.74 | 6.92 | 3.74,12.81 | 3.81 | 2 | 0.15 | 5.31 | 3.00, 9.39 | 1.14 | 2 | 0.56 |
| **Region** | 8.02 | 4.45, 14.47 | 2.88 | 4 | 0.58 | 7.7 | 4.29, 13.81 | 1.24 | 4 | 0.87 | 6.09 | 3.57, 10.38 | 3.16 | 4 | 0.53 |
| **Highest qualification of respondent^b^** | 8.10 | 4.48, 14.66 | 4.65 | 4 | 0.32 | 7.09 | 4.03, 12.47 | 4.61 | 4 | 0.33 | 6.16 | 3.59, 10.56 | 0.75 | 4 | 0.94 |
| **Age of respondent** | 8.76 | 4.72, 16.24 | 0.32 | 2 | 0.85 | 7.59 | 4.222, 13.63 | 0.72 | 2 | 0.70 | 6.09 | 3.58, 10.38 | 2.36 | 2 | 0.31 |
| **BMI of respondent^c^** | 7.59 | 3.84, 14.97 | 1.69 | 2 | 0.43 | 6.31 | 3.27, 12.18 | 0.67 | 2 | 0.72 | 6.02 | 3.21, 11.29 | 0.81 | 2 | 0.67 |
| **Household size** | 7.78 | 4.35, 13.93 | 1.44 | 2 | 0.49 | 7.55 | 4.22, 13.51 | 2.12 | 2 | 0.35 | 6.13 | 3.58, 10.50 | 0.28 | 2 | 0.87 |

Notes: ^a^ missing data for 49 respondents; ^b^ missing data for 88 respondents

**Table S10**. Child-child consumption correlation

|  | | **N** | **Sugar** | | | **Low or no-sugar/diet** | | | **Juice** | | |
| --- | --- | --- | --- | --- | --- | --- | --- | --- | --- | --- | --- |
|  |  |  | **OR** | **95% CI** | **p value** | **OR** | **95% CI** | **p value** | **OR** | **95% CI** | **p value** |
| **Children 0-6 years** | Crude odds of consuming beverages if sibling 7-12 does | 99 | 102.81 | 5.80, >1800 | <0.001 | 15.84 | 5.41, 46.37 | <0.001 | 26.07 | 7.94, 85.57 | <0.001 |
|  | Crude OR of consuming beverages if sibling 13-18 does | 31 | *perfect correlation ^a^* | | | 11.67 | 2.13, 6.04 | 0.002 | 3.42 | 0.75, 15.67 | 0.10 |
| **Children 7-12 years** | Crude OR of consuming beverages if sibling 13-18 does | 124 | 22.33 | 8.60, 58.01 | <0.001 | 120.75 | 15.20, 959.10 | <0.001 | 18.59 | 7.27, 47.50 | <0.001 |

Notes: ^a^ In all 31 households with children 0-6 and 13-18, all children 0-6 consumed sugary drinks if they had siblings 13-18 consumed sugary drink
